# ADRD RISK REDUCTION HEALTH COACHING

**DOI:** 10.1093/geroni/igae098.1688

**Published:** 2024-12-31

**Authors:** Faika Zanjani, Cate Hawks, Kennedy O’Donnell, Brian Berman, Lana Sargent

**Affiliations:** Virginia Commonwealth University, Richmond, Virginia, United States; Virginia Commonwealth University, Richmond, Virginia, United States; Virginia Commonwealth University, Richmond, Virginia, United States; Virginia Commonwealth University, Richmond, Virginia, United States; Virginia Commonwealth University, Richmond, Virginia, United States

## Abstract

**Objectives:**

The development of the Richmond Brain Health Initiative (RBHI) was guided by the need to address local brain health service gaps to improve Alzheimer’s/Dementia (ADRD) health disparities in racially diverse communities. This paper focuses on the ADRD Risk Reduction Health Coaching program component.

**Methods:**

The program is currently in implementation, focused on recruiting diverse older adults living in the Richmond, VA metro area, with the following risk factors: diabetes, hypertension, cardiovascular disease, or subjective cognitive decline. Weekly lifestyle telephone health coaching for 12 weeks was offered, followed by a monthly (6-month) maintenance stage. Health coaching calls last an average of 15-30 minutes per session. The intervention protocol target lifestyle risk via education, learning objectives, personal risk assessment, attitudes, and lifestyle behavioral change goal setting.

**Results:**

The current study sample (n=49) is 96% 60+ years old (n=38), 38% African American/Black (n=18), 6% Hispanic (n=3), 70% female (n=33), 11% military veterans (n=5), 53% live alone (n=25). Preliminary mean differences indicate improvement in ADRD knowledge: baseline mean = 25.8, 12-week = 28.6; Physical Activity: baseline mean = 1.50 hours/week, 12-week = 3.00 hours/week; Social Activity: baseline mean = 2.07 hours/week, 12-week = 2.57 hours/week; Cigarettes: baseline mean = 0.62/day, 12-week = 0.30/day; Alcoholic Drinks: baseline mean = 1.50/week, 12-week = 1.10/week; and Medication adherence: baseline = 86%, 12-week = 100% adherence. CONCLUSIONS: This study showcases a model for reducing ADRD risk and promoting brain health in racially diverse communities by improving access to ADRD community services.

